# Calcium-Binding Proteins S100A8 and S100A9: Investigation of Their Immune Regulatory Effect in Myeloid Cells

**DOI:** 10.3390/ijms19071833

**Published:** 2018-06-21

**Authors:** Jianxin Yang, Jacqueline Anholts, Ulrike Kolbe, Janine A. Stegehuis-Kamp, Frans H. J. Claas, Michael Eikmans

**Affiliations:** Department of Immunohematology and Blood Transfusion, Leiden University Medical Center, 2333 ZA Leiden, The Netherlands; j.yang@lumc.nl (J.Y.); J.D.H.Anholts@lumc.nl (J.A.); Ulrike.Kolbe@med.uni-heidelberg.de (U.K.), J.A.Stegehuis-Kamp@lumc.nl (J.A.S.-K.); F.H.J.Claas@lumc.nl (F.H.J.C.)

**Keywords:** calcium-binding proteins, monocytes subsets, S100A9 subsets, immune regulation

## Abstract

High expression levels of the calcium-binding proteins S100A8 and S100A9 in myeloid cells in kidney transplant rejections are associated with a favorable outcome. Here we investigated the myeloid cell subset expressing these molecules, and their function in inflammatory reactions. Different monocyte subsets were sorted from buffy coats of healthy donors and investigated for S100A8 and S100A9 expression. To characterize S100A9high and S100A9low subsets within the CD14+ classical monocyte subset, intracellular S100A9 staining was combined with flow cytometry (FACS) and qPCR profiling. Furthermore, S100A8 and S100A9 were overexpressed by transfection in primary monocyte-derived macrophages and the THP-1 macrophage cell line to investigate the functional relevance. Expression of S100A8 and S100A9 was primarily found in classical monocytes and to a much lower extent in intermediate and non-classical monocytes. All S100A9+ cells expressed human leukocyte antigen—antigen D related (HLA-DR) on their surface. A small population (<3%) of CD14+ CD11b+ CD33+ HLA-DR− cells, characterized as myeloid derived suppressor cells (MDSCs), also expressed S100A9 to high extent. Overexpression of S100A8 and S00A9 in macrophages led to enhanced extracellular reactive oxygen species (ROS) production, as well as elevated mRNA expression of anti-inflammatory *IL-10*. The results suggest that the calcium-binding proteins S100A8 and S100A9 in myeloid cells have an immune regulatory effect.

## 1. Introduction

The S100 calcium-binding proteins A8 and A9 (S100A8 and S100A9), also known as migration inhibitory factor-related proteins 8 (MRP8) and 14 (MRP14), are abundantly expressed in myeloid cells, such as circulating monocytes and neutrophils. Their level of expression can be used as a biomarker of inflammation in bacterial infections and autoimmune diseases [1,2,3,4,5]. At the protein level S100A8 and S100A9 can form a heterodimeric complex, which is called calprotectin and which has antimicrobial effects [6,7,8]. Extracellular S100A8 and S100A9 proteins bind to Toll-like receptor 4 and the receptor for advanced glycation end products (RAGE) to trigger NF-κB activation and production of proinflammatory cytokines and chemokines [9,10].

In contrast, several reports have provided evidence that S100A8 and S100A9 can exert anti-inflammatory effects: this was shown in lipopolysaccharides (LPS)-induced endotoxemia and autoimmune myocarditis [11,12]. Intracellular S100A9 can regulate adaptive immunity by inducing accumulation of myeloid-derived suppressor cells (MDSCs) in tumor-bearing individuals [13,14,15]. MDSCs are able to suppress T cell responses. Chen and colleagues showed that S100A9 inhibits the differentiation of dendritic cells and macrophages and induces accumulation of MDSCs through elevated reactive oxygen species (ROS) production [14]. Sinha and colleagues reported that S100A8/A9 binds to glycoprotein receptors on MDSCs and promotes their migration and accumulation [15]. S100A9 was proposed as a novel marker of human monocytic MDSCs [16].

The role of myeloid cells in allograft rejection has been increasingly appreciated [17,18,19]. We found in human kidney transplants that acute rejection episodes with high tissue expression of *S100A8* and *S100A9* have a more favorable long-term outcome than rejections with low expression [20,21], suggesting that the S100 proteins exert beneficial immune effects. Double immunofluorescence on tissue biopsies showed that S100A9 largely co-localized with CD68 and HLA-DR, but that only a minority of S100A9+ cells expressed the macrophage type 2 marker CD163. This suggests that S100A9+ cells infiltrating the graft represent a distinct macrophage subset that potentially can interact with T cells through their surface HLA class II molecules. Furthermore, both in peripheral blood mononuclear cells (PBMC) and biopsies, we observed correlations of *S100A9* expression with the expression of *CD11b* and *CD33* [21]. The combination of high CD11b and CD33 and low HLA-DR is used by flow cytometry to distinguish MDSCs [22]. MDSCs have been observed to accumulate in kidney transplant recipients, and they were able to induce expansion of regulatory T cells in vitro [23,24]. Furthermore, patients with high numbers of MDSCs in their blood at time of acute transplant rejection had a favorable graft outcome [24].

Based on previous findings we hypothesize that S100A9+ myeloid cells have distinct immune regulatory properties. In the current study, we phenotypically characterized monocytes that differentially expressed S100A8 and S100A9, and identified a functional role of these calcium-binding proteins in macrophages.

## 2. Results

### 2.1. S100A9 is Mostly Expressed in CD14-Positive (Classical) Monocytes

**Figure 1:**
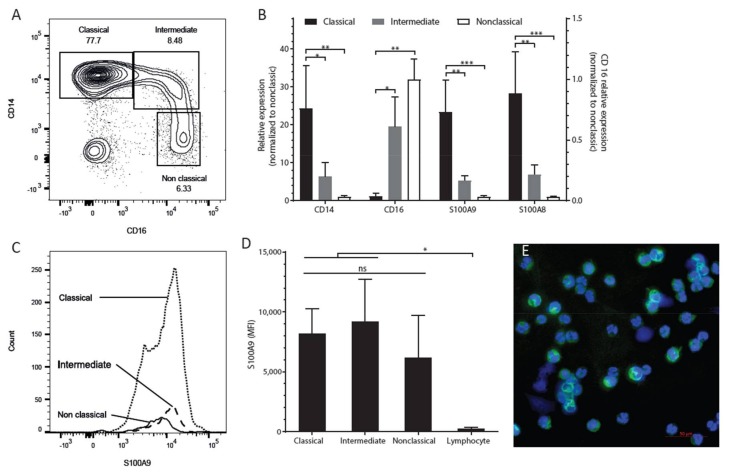
S100A9 expression is highest in CD14+ classical monocytes. (**A**) Classical, intermediate, and nonclassical monocytes subsets were sorted based on CD14 and CD16 expression using FACS; (**B**) The relative expression of S100A9 in the classical subset was 20-fold higher than that in the non-classical subset; (**C**) The representative FACS histogram plot showed that S100A9 expression in the three monocyte subsets overlapped with each other; (**D**) The median fluorescence intensity (MFI) of S100A9 in the classical subset was approximately twice as high than that in the non-classical subset; (**E**) The cytospin results showed that the fluorescence intensity varied greatly between individual cells within the CD14+ monocyte population; scale bar: 50 µm. The differences were tested by one-way ANOVA with Tukey’s multiple comparison tests. Data are expressed as means ± SD of at least three biological replicates. * *p* < 0.05, ** *p* < 0.01, *** *p* < 0.001.

S100A9 expression levels were assessed in three monocyte subsets, designated as classical (CD14+ CD16−), intermediate (CD14+ CD16+), and non-classical (CD14− CD16+) monocytes (Figure 1A). Messenger RNA analysis of *CD14* and *CD16* in the three sorted populations verified the sorting strategy (Figure 1B). Expression of S100A9 was most abundant in the classical monocytes (Figure 1B), which encompassed at least 75% of the total monocyte population (Figure 1A). Protein expression of S100A9 by flow cytometry was seen in all three monocyte subsets, and it was higher than that seen in lymphocytes (Figure 1C,D). The median fluorescence intensity (MFI) of S100A9 in classical and intermediate monocytes was approximately twice as high as that of non-classical monocytes (Figure 1D). The results show that S100A9 is mostly expressed in CD14-positive monocytes.

### 2.2. S100A9 Expression Varies within the CD14+ Monocyte Population

Next, we tested whether there is variation in S100A9 expression within the CD14+ monocyte population. For this, we subjected CD14+ enriched cells to cytospin analysis of S100A9 protein. The fluorescence intensity varied greatly between cells (Figure 1E). Similarly, the fluorescence-activated cell sorting (FACS) plot showed a wide range of S100A9 expression within the CD14+ classical monocytes (Figure 1C).

### 2.3. Both HLA-D-Positive Monocytes and Myeloid Derived Suppressor Cells Express S100A9

**Figure 2:**
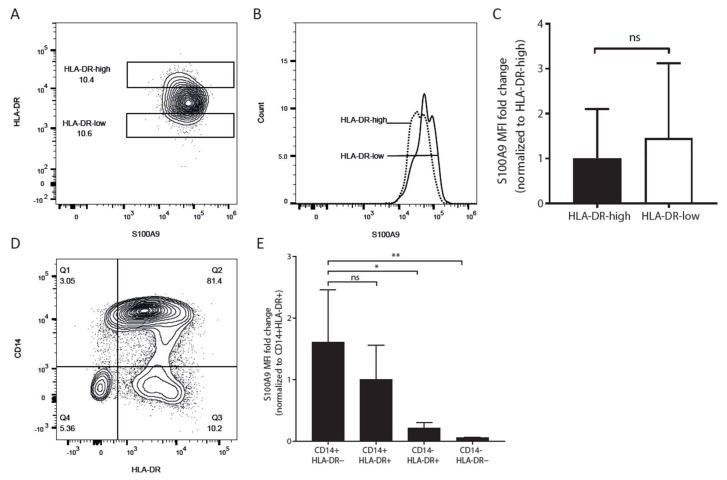
S100A9-positive monocytes express HLA-DR. Peripheral blood mononuclear cells (PBMC) were stained for CD14, HLA-DR, and S100A9, and were gated on CD14+ cell populations. (**A**) Representative FACS plot showing that CD14+ monocytes express both HLA-DR and S100A9. Gates were set to the upper and lower 10% of HLA-DR expression for the CD14+ monocytes, to further study subsets; (**B**,**C**) S100A9 expression was not significant between HLA-DR-high and HLA-DR-low subsets, although, S100A9 was slightly higher in the latter subset. Significant differences were calculated by *t*-test; (**D**) Representative FACS plot showing expression of CD14 and HLA-DR in gated CD33+ myeloid cells; (**E**) Monocytic MDSC (CD14+ HLA-DR−) subsets showed slightly higher expression of S100A9 than CD14+ HLA-DR+ subsets, but this was not significant. The differences were tested by one-way ANOVA with Tukey’s multiple comparison tests. All data are expressed as means ± SD of four different experiments. * *p* < 0.05, ** *p* < 0.01.

To investigate whether S100A9-positive monocytes express HLA-DR on their surface and are potentially able to interact with CD4+ T cells, we analyzed S100A9 and HLA-DR expression by FACS on PBMC from healthy donors. HLA-DR and S100A9 were co-expressed in CD14+ monocytes (Figure 2A). HLA-DR-low monocytes showed slightly higher expression of S100A9 than HLA-DR-high monocytes in healthy donors (Figure 2B,C). The results show that all S100A9-positive monocytes express HLA-DR on their surface.

We further observed that MDSCs, which are characterized as CD14+ CD11b+ CD33+ HLA-DR− and constitute only a small percentage of the total monocyte population (Figure 2D), showed an even higher S100A9 expression than the CD14+ HLA-DR+ subset, although this difference was not significant (Figure 2E).

### 2.4. Phenotypic Characterization of Cytokine Expression Profiles in S100A9high and S100A9low Monocytes

**Figure 3:**
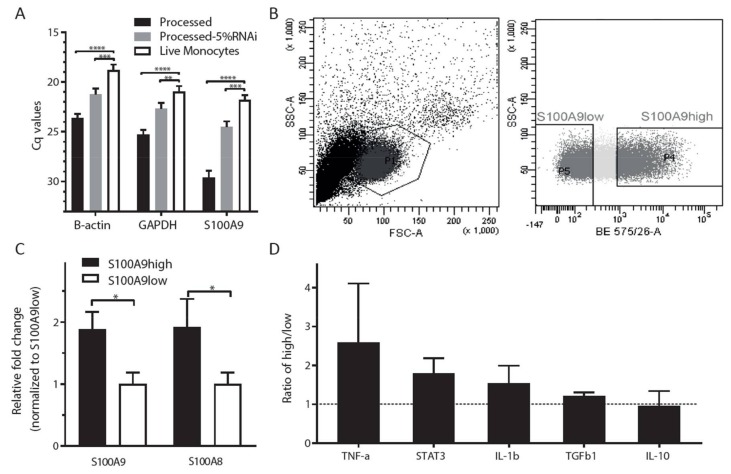
S100A9high and S100A9low subsets of classical monocytes do not differ in their expression of immune-related cytokines. (**A**) The addition of increased RNAse inhibitor concentration (5%) to lysis buffer minimizes the extent of RNA degradation (delta Cq < 34). Differences were tested by one-way ANOVA with Tukey’s multiple comparison tests; (**B**) Monocytes were sorted into two subsets based on the S100A9 protein level: S100A9high and S100A9low; (**C**) Relative gene expression of S100A9 from FACS sorted cell populations based on S100A9 protein levels. The S100A9high subset expressed significantly higher S100A9 mRNA levels than the S100A9low subset. The difference was tested by *t*-test; (**D**) The relative mRNA expression ratio of pro- and anti-inflammatory cytokines was not significantly different between S100A9 high and S100A9 low subsets. The dashed line indicates relative mRNA expression ratio between S100A9 high and S100A9 low subsets is one. The differences were tested using one sample *t*-test. All data are expressed as means ± SD of three different experiments. * *p* < 0.05, ** *p* < 0.01, *** *p* < 0.001, **** *p* < 0.0001.

To test whether S100A9high and S100A9low monocytes differ phenotypically in their cytokine expression profile, we combined intracellular S100A9 FACS staining with mRNA analysis of sorted cell populations. Since fixation and intracellular FACS staining disturbs the RNA quality inside the cells, the assay needed to be optimized first. Processed (fixed and intracellular stained) cells showed significantly reduced qPCR signals (as indicated by higher Cq values) compared to live cells (Figure 3A). Application of a buffer containing 5% RNase inhibitor greatly improved RNA quality, as detected by a significant decrease in the Cq value by qPCR (Figure 3A).

The monocytes were then sorted into two subsets based on their S100A9 protein level: S100A9high and S100A9low (Figure 3B). S100A9high subsets expressed significantly higher S100A9 mRNA levels than S100A9low subsets (Figure 3C). Several cytokines and growth factors were measured at the RNA level in the S100A9high and S100A9low subsets, but we did not observe significant differences between the subsets in expression levels of acute phase proteins *TNFα*, *IL-1β*, and of anti-inflammatory cytokines *TGFβ1* and *IL-10* (Figure 3D).

### 2.5. Sorting of Viable Cells: Application of SmartFlare

To sort viable monocytes expressing S100A9 for further functional analysis, SmartFlare RNA detection probes were applied. The probes, attached to inert nanogold particles, are taken up by the cells and emit a fluorescent signal upon binding to their target S100A9 mRNA transcripts within the cells. Isolated CD14+ monocytes were incubated with different amounts of SmartFlare probes for 16 h. The scrambled control probe, which does not recognize any mRNA sequence within the cells, generated a strong fluorescence signal indicating a high level of background staining with all tested concentrations (Figure S1A). Monocytes incubated with the SmartFlare probes were sorted into three groups based on the Cy5 fluorescence intensity (Figure S1B), and the level of S100A9 expression was then validated in each of these three subsets by qPCR and intracellular staining using anti-S100A9 monoclonal antibody. The Cy5high monocyte subset showed no difference of *S100A9* mRNA expression in comparison to the Cy5intermediate and Cy5low subsets (Figure S1C). The intracellular S100A9 protein expression was comparable among the three sorted cell subsets (Figure S1D). Therefore, functional analysis of the monocyte subsets that differentially express S100A9 could not be performed because of unreliable detection by the SmartFlare probes.

### 2.6. Overexpression of S100A8 and S100A9 in Monocyte-Derived Macrophages Leads to Increased ROS Production and Elevated IL-10 mRNA Expression

**Figure 4:**
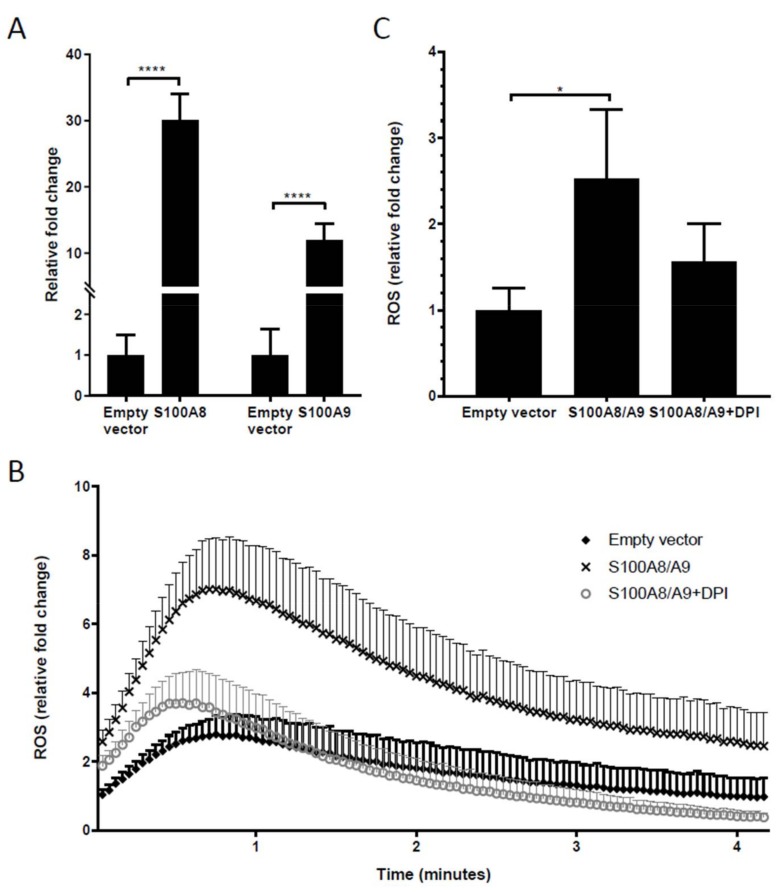
Overexpression of S100A8/A9 in monocyte-derived macrophage increases reactive oxygen species (ROS) production. (**A**) The mRNA level in monocyte-derived macrophages after transfection was significantly higher than that in the empty plasmid control. The differences were tested by *t*-tests; (**B**) After phorbol 12-myristate 13-acetate (PMA) activation, the transfected cells were placed immediately in a luminometer to measure their ROS production real-time every 2.5 s. Data are expressed as means ± SEM of at least four different experiments; (**C**) The peak of ROS production, assessed at the tenth timepoint (25 s) was significantly higher in the S100A8/A9-overexpressed macrophages compared to cells transfected with the empty plasmid. Inhibition of NADPH oxidase activity by diphenylene iodonium (DPI) blocked the ROS production. The differences were tested by one-way ANOVA with Tukey’s multiple comparison tests. All data are expressed as means ± SD of at four different experiments. * *p* < 0.05, **** *p* < 0.0001.

To investigate the functional consequence of intracellular S100A9, and its counterpart S100A8, a plasmid containing the full S100A8 and S100A9 sequences was transfected into monocyte-derived macrophages. Transfection led to a 20–40-fold overexpression of S100A8 and S100A9 mRNA (Figure 4A). Overexpression of S100A8 and S100A9 in the monocyte-derived macrophages led to a threefold increase of ROS production (Figure 4B,C). The inhibition of nicotinamide adenine dinucleotide phosphate (NADPH) oxidase activity by diphenylene iodonium (DPI) normalized the elevated ROS levels in the transfected macrophages, showing that S100A8/S100A9-induced ROS production was exerted through NADPH activation. To examine the effect of ROS-producing macrophages on T cells, macrophages were co-cultured with allogeneic T cells. Unfortunately, transfection of the empty control plasmid already led to decreased induction of T cell stimulation compared to untransfected macrophages, suggesting that the transfection procedure interferes with the macrophages’ T cell stimulating capacity. 

**Figure 5:**
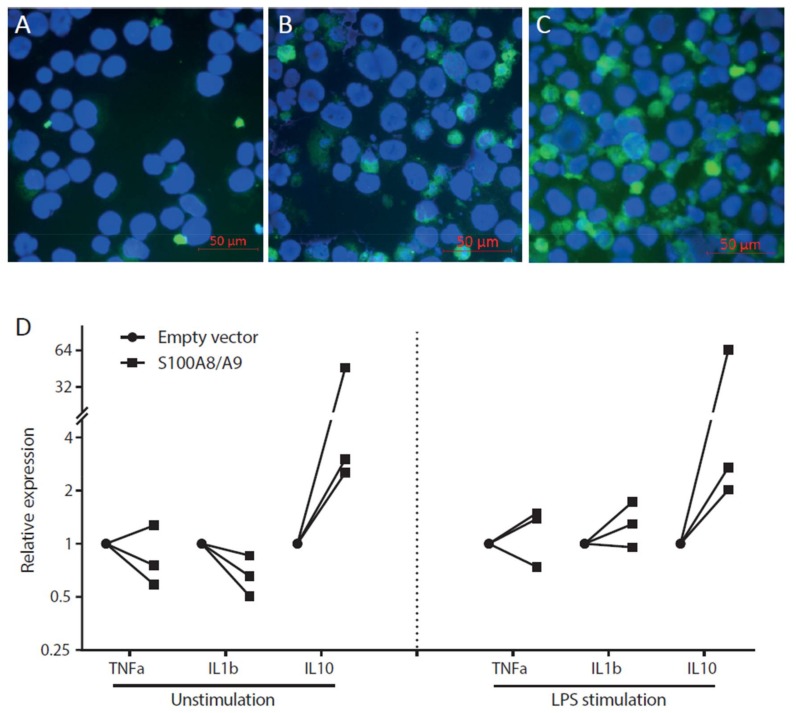
Overexpression of S100A8/A9 in macrophages leads to increased IL-10 expression. (**A**–**C**) Validation of transfection efficiency of the THP-1 macrophage cell line. The following cytospins were inspected by immunofluorescence for green light emission: (**A**) non-transfected cells; (**B**) cells transfected with an empty plasmid; (**C**) and those with a GFP-containing plasmid. Scale bar: 50 µm. The results show high transfection efficiency in the cells; (**D**) Overexpression of S100A8 and S100A9 leads to a consistent increase of IL-10 expression, but not of TNFα and IL-1β. This was observed in both unstimulated cells (**left** panel) and LPS–stimulated cells (**right** panel). Data are shown as boxplots (median, upper value, lower value) representing three different experiments.

We next overexpressed the S100 calcium-binding proteins in THP-1 macrophages to study effects on cytokine synthesis by these cells. To first evaluate the transfection efficiency of the cells, a green fluorescent protein (GFP)-containing plasmid was transfected for 24 h. The high proportion of GFP-positive cells indicates a high transfection efficiency (Figure 5C). We did observe that transfection with an empty vector already caused slight changes compared to non-transfected cells both with respect to GFP signals (Figure 5A,B) and cell viability, which led us to compare the differences between the empty vector and S100A8/A9 transfection conditions. Overexpression of S100A8 and S100A9 in macrophages, both in unstimulated cells and in cells that were stimulated by LPS after the transfection, led to a consistent increase (2-fold to 64-fold) in IL-10 mRNA expression compared to the empty vector transfection condition (Figure 5D). At the same time, expression of pro-inflammatory cytokines TNFα and IL-1β did not change. Excreted cytokines in the supernatants were assessed by Luminex assays, but only TNFα could be detected, which showed no difference between the S100A8/A9 overexpressed cells and the control conditions.

## 3. Discussion

Calcium-binding proteins S100A8 and S100A9 are involved in various inflammatory disorders. Relatively high levels of S100A8 and S100A9 in kidney biopsies have a beneficial effect on long-term graft outcome, independent of the extent of myeloid cell infiltration. In this study, we phenotypically characterized the monocyte subsets that differentially express S100A8 and S100A9. We further showed that overexpression of S100A8 and S100A9 in macrophages leads to enhanced ROS production and IL-10 expression.

Monocytes have been documented to consist of three main cell populations based on the expression level of CD14 and CD16: classical, intermediate, and nonclassical [25,26,27]. At the mRNA level, *S100A8* and *S100A9* were abundantly expressed in the classical monocyte subset [26,28,29]. At the protein level, the three subsets were more similar in their expression, with a small trend for the non-classical monocytes to express the lowest levels. In line with an earlier study where proteomic and transcriptomic methods were used, CD16-negative monocytes had higher mRNA and protein levels of S100A9 than the CD16-positive subsets [29]. We demonstrated a heterogeneous expression of S100A9 within the CD14+ monocyte population, which was the basis for the hypothesis that monocytes with high levels of S100A9 exert distinct immune regulatory effects.

As the expression of S100A9 in the CD14+ HLA-DR-low subset was even slightly higher than that in the HLA-DR-high subset, it is clear that HLA-DR is not an appropriate cell surface marker for sorting monocytes that differently express S100A9 protein. Consistent with our earlier findings in transplant tissue [21], S100A9 and HLA-DR are co-expressed in peripheral CD14+ monocytes. Furthermore, the monocytic MDSC subset (CD14+ CD11b+ CD33+ HLA-DR−), which represented a tiny fraction (<3%) of the total myeloid population in our healthy donor material, showed an even higher S100A9 expression than the CD14+ HLA-DR+ subset, although this difference was not significant. It does support the earlier findings of S100A9 as both a marker and an inducer of MDSCs. On the basis of previous publications [23], it is expected that in inflammatory situations such as post-transplant conditions, the fraction of MDSCs, and thereby S100A8 and S100A9, rises considerably. The current findings further show that not a single subset, but multiple ones, express the S100 molecules to a relatively high extent.

We investigated S100A9high and S100A9low monocytes, based on intracellular FACS staining, for cytokine expression. Recently, a method for the isolation of high quality RNA from cells following fixation, intracellular staining, and FACS sorting by the use of buffers containing RNase inhibitors has been described [30]. However, when following that protocol we observed that the process of intracellular staining still led to reduced RNA quality compared to live, unfixed monocytes. A possible explanation for this discrepancy might be the fact that monocytes contain more ribonuclease A family member 2 (RNASE2) than the human embryonic stem cells, as found by microarray analysis [31], in which the RNA quality was improved by increasing the RNase inhibitor concentration [30]. Nevertheless, when normalized to reference gene signals to correct for input and quality differences, we could verify significantly increased *S100A9* mRNA expression in the FACS S100A9high subset compared to the S100A9low subset, which gave confidence with respect to the reliability of the results for the other transcripts studied.

SmartFlare probes have been suggested as suitable tools to detect mRNA in single living cells. Several studies showed successful detection of mRNA using SmartFlare or NanoFlare probes [32,33,34,35]. In contrast to these publications, in our study the mRNA levels reflected by SmartFlare fluorescence intensity could not be validated by qPCR and flow cytometry. The *S100A9* expression levels were as high as the reference gene (*GAPDH*) and were certainly above the SmartFlare detection limit (Cq < 34). Consistent with our findings, Maria and colleagues demonstrated that SmartFlare fluorescence intensity does not correlate with the level of target transcript, but depends on the efficiency of probe accumulation [36]. We found that the SmartFlare fluorescence intensity positively correlated with the forward scatter value, which reflects cell size. Levy and colleagues showed by electron microscopy that the nanoparticle probes remain in intracellular compartments and do not reflect the level of mRNA transcripts in the cytoplasm [37]. Mirkin and colleagues, who developed the NanoFlare probes, confirmed that spherical nucleic acid (SNA) localize in late endosomes. They also proposed that the fluorescence signal detected may be due to disassembly of SNA and degradation by the DNaseII [38]. This may explain why we observed high background levels in the scrambled probes.

To investigate the functional effect of S100A8 and S100A9, we overexpressed both molecules in primary monocyte-derived macrophages. We did observe that this led to significantly increased ROS production, which was similar to what was found in our previous study using the established, secondary THP-1 macrophage cell line [21]. Through this mechanism, the S100 calcium-binding proteins may exert anti-inflammatory effects, and thereby be beneficial in reducing tissue damage after transplantation. In contrast to THP-1 cells, which lack crucial co-stimulation molecules, monocyte-derived macrophages are able to stimulate (allogeneic) T cells in a mixed lymphocyte culture. When present in the immune synapse during interaction of macrophages with T cells, the extracellular ROS may negatively affect T cell activation and proliferation [39]. We observed that the transfer of a large plasmid (~7000 base pairs) into the cells negatively affects cell viability and T cell stimulating potential, which unfortunately held us back from performing mixed lymphocyte cultures between transfected macrophages and T cells. Interestingly, in relation to the link between S100A8/A9 and ROS, the MDSC population in our study was found to express the highest levels of S100A8 and S100A9. MDSCs have been shown to exert T cell inhibiting effects in individuals with a tumor and in those with inflammatory conditions, such as those seen after transplantation [13,14,23,40]. S100A9 can be involved, through STAT3 and ROS, in inducing accumulation of MDSCs [13,14]. We did observe that transfection with the empty vector caused some changes compared to non-transfected cells with respect to GFP signals and cell viability, as discussed above. Indeed, it has been described before that green fluorescence production may result from the mere presence of a plasmid and from the use of transfection reagents [41]. These observations led us to compare the differences between the empty vector and S100A8/A9 transfection conditions. We found that S100A8/A9-transfected macrophages demonstrate higher expression of the anti-inflammatory cytokine *IL-10*, but not of pro-inflammatory cytokines *TNFα* and *IL-1β*. Despite the consistent *IL-10* mRNA increase in all experiments, a statistically significant difference was not obtained between cells transfected with S100A8/A9 and those transfected with empty vectors, probably because of the variation in the range of increase between experiments. Despite our efforts using protein screening in the supernatant at 24 h after transfection, IL-10 protein could not be detected. Hence, it cannot be stated with certainty that IL-10 is a significant mediator in the induction of immune regulation. Especially in the light of the presence of HLA-DR on S100A9-positive cells, both ROS and IL-10 may represent parts of a pathway through which the S100 molecules trigger the induction of regulatory T cells and inhibition of effector T cells, thereby limiting or preventing a detrimental immune response to the graft.

In summary, the results suggest that calcium-binding proteins S100A8 and S100A9 in myeloid cells have an immune regulatory effect, which may explain the previously observed beneficial effect of the local S100A8 and S100A9 expression on kidney graft survival [20,21].

## 4. Materials and Methods

### 4.1. Reagents

The following reagents were used for the study: phosphate-buffered saline (PBS, 10×) pH 7.4 (ThermoFisher-Ambion, Cat. No. AM9625, Vilnius, Lithuania), distilled water (DNase/RNase free, Gibco, Cat. No. 10977-035), paraformaldehyde (PFA) (EM Grade, Purified, Electron Microscopy Sciences, Hatfield, PA, USA), Saponin (Sigma-Aldrich, Saint Louis, MO, USA), recombinant ribonuclease inhibitor (ThermoFisher-Invitrogen, Cat. No. 10777-019, Carlsbad, CA, USA), bovine serum albumin (BSA) fraction V-molecular biology grade (Gemini bio-products, Cat. No. 700-106P, Burgess Hill, UK), PE mouse anti-human S100A9 Clone 1H9 (BD bioscience-Pharmingen, Cat. No. 565793, San Diego, CA, USA), rabbit-anti-human S100A9 (ab92507, Abcam, Cambridge, UK), goat-anti-rabbit IgG-AF488 (A11008, Invitrogen, Bleiswijk, The Netherlands), RPMI1640 (Gibco, Grand island, NY, USA), fetal calf serum (FCS), penicillin, streptomycin, L-glutamine (Gibco, Invitrogen, Carlsbad, CA, USA), diphenyleneiodonium (DPI; Sigma-Aldrich, D2926, Saint Louis, MO, USA), GM-CSF (Thermo-Fisher, PHC2013-1MG, Carlsbad, CA, USA), mouse anti-human antibody against CD14, CD16, and HLA-DR (all BD Bioscience).

### 4.2. Monocyte Purification

Written informed consent was obtained from donors for use of buffy coats for scientific purposes. Peripheral blood mononuclear cells (PBMC) were isolated by Ficoll-Hypaque density gradient centrifugation from buffy coats of anonymous healthy donors (Sanquin blood bank, Leiden, the Netherlands). CD14+ monocytes were isolated from PBMC by positive selection using human CD14 microbeads (Miltenyi biotec GmbH, Bergisch Gladbach, Germany) according to the manufacturer’s instructions.

### 4.3. Monocyte Subsets FACS Sorting

Purified PBMC were stained with antibodies against CD14 and CD16 in 100 μL of FACS buffer. After washing twice, three monocyte subsets were sorted using FACSAria flow cytometer according to the CD14 and CD16 expression level. Sorted subsets were pelleted and lysed for RNA isolation.

### 4.4. Measurement of S100A9 in Monocytes Using the Cytospin Method

CD14 microbeads-enriched monocytes (positive selection) were washed with PBS and resuspended at a concentration of 0.5 × 10^6^ cells/mL. Three drops of cell suspension were transferred onto a glass slide using a cytospin 4 cytocentrifuge (Thermo Scientific, Waltham, MA, USA) at room temperature. Cells were fixed using cold acetone for 10 min and washed three times. Cells were incubated with a 1000-times-diluted rabbit-anti-human S100A9 antibody for 1 h at room temperature. After washing, slides were incubated with 1:200 diluted goat-anti-rabbit IgG-AF488 for 30 min. Images were acquired on a fluorescence microscope (Axioskop 40, Carl Zeiss, Jena, Germany) using digital imaging software (ZEN 2.3 lite, Carl Zeiss, Jena, Germany).

### 4.5. Intracellular Staining and FACS Sorting

Intracellular staining procedures, followed by RNA extraction and qPCR, were mainly based on a previous study [30]. In brief, one million CD14+ monocytes were fixed and permeabilized with 4% PFA and 1% saponin in 250 μL of PBS, supplemented with 1–5% RNase inhibitor for 30 min at 4 °C. Cells were pelleted and washed once using 1 mL of wash buffer (0.2% BSA, 0.1% saponin, 2.5% RNase inhibitor in PBS) at 4 °C. Cells were incubated for 30 min at 4 °C with 5 μL of mouse anti-human S100A9 antibody in 50 μL of staining buffer (1% BSA, 0.1% saponin, 5% RNase inhibitor in PBS). Cells were washed twice with 1 mL of wash buffer and resuspended in sorting buffer (0.5% BSA, 5% RNase inhibitor in PBS). Cells were sorted into two subsets based on S100A9 protein expression using a FACSAria flow cytometer (BD Biosciences). Intracellular staining used for flow cytometric analysis were performed using the BD Cytofix/Cytoperm kit (BD Biosciences, Cat. No. 554714)

### 4.6. RNA Isolation and cDNA Synthesis

After flow cytometric sorting, the cell suspension was centrifuged at 3000*g* for 5 min at 4 °C and the supernatant was removed. Total RNA from intracellular stained cells was isolated using the RecoverAll Total Nucleic Acid Isolation Kit for FFPE (Ambion, AM1975, Carlsbad, CA, USA), by incubating with 100 μL of protease digestion buffer for 3 h at 50 °C. The other steps in the RNA isolation procedure were carried out according to the manufacturer’s instructions. When working with unstained, intact cells, total RNA was extracted using the NucleoSpin miRNA kit (Macherey-Nagel, Düren, Germany). Complementary DNA synthesis was performed as described previously [21,42].

### 4.7. Real-Time Quantitative PCR

Primer sequences for quantitative PCR are provided in Table S1. To prevent amplification of genomic DNA, reverse and forward primers were designed to target separate exons, spanning at least one intron with a size of 800 bp or more. All primer sets were tested on control cDNA, and PCR efficiencies were between 90% and 110%. Relative gene expression levels were normalized to the geometric mean of the reference genes β-actin and glyceraldehyde 3-phosphate dehydrogenase (GAPDH).

### 4.8. Overexpression of S100A8/A9 and Measurement of ROS and Cytokines

To allow differentiation into macrophages, CD14+ monocytes were cultured in the presence of 5 ng/mL GM-CSF for 5 days. Monocyte-derived macrophages were seeded at 1.5 × 10^6^ per well in 6-well plates. After 5 days, transfection mixture containing 20 μL of Lipofectamine 2000 (Thermo Fisher) and 7.5 μg plasmid was added dropwise to the wells. After 5–6 h the medium was replaced by fresh complete roswell park memorial institute (RPMI) medium and incubated for another 24 h. The extracellular ROS production by the cells was measured as described previously [21].

The THP-1 macrophage cell line was transfected with similar amounts of plasmid as described above, and incubated for 24 h. Cells were washed and resuspended for incubation for another 24 h in complete medium, either containing no stimulants or containing 100 ng/mL LPS. The cells were then pelleted and lysed for RNA isolation.

### 4.9. Statistical Analyses

Statistical analyses were performed using SPSS statistics 23 and GraphPad Prism 7.02. All experiments were performed at least three times. The statistical difference between the two groups was calculated by *t*-test, and the difference between the two groups was calculated by one-way ANOVA with Tukey’s multiple comparison tests.

## Supplementary Materials

Supplementary materials can be found at http://www.mdpi.com/1422-0067/19/7/1833/s1.
