# Supplementary material for: Calcium-Binding Proteins S100A8 and S100A9: Investigation of Their Immune Regulatory Effect in Myeloid Cells
Source: Int J Mol Sci. 2018 Jun 21;19(7):1833. doi: 10.3390/ijms19071833 (PMC6073713; doi:10.3390/ijms19071833)
Supplement: Supplementary file 1 [file ijms-19-01833-s001.pdf]

Table S1. Primer sequences used for qPCR.

Genes	Forward Primer (5′-3′)	Reverse Primer (5′-3′)
B-actin	ACCACACCTTCTACAATGAG	TAGCACAGCCTGGATAGC
GAPDH	ACCCACTCCTCCACCTTTGAC	TCCACCACCCTGTTGCTGTAG
IL-10	GCGCTGTCATCGATTTCTTCC	GTAGATGCCTTTCTCTTGGAGCTTA
IL-1b	TGGCTTATTACAGTGGCAATG	GTGGTGGTCGGAGATTCG
S100A8	GGGAATTTCCATGCCGTCT	CCTTTTTCCTGATATACTGAGGACACT
S100A9	CAGCTGGAACGCAACATAGA	TCAGCTGCTTGTCTGCATTT
STST3	CACGCCTTCTACAGACTG	CATCCTGGAGATTCTCTACC
TGF-b1	CCCAGCATCTGCAAAGCTC	GTCAATGTACAGCTGCCGCA
TNF-a	CCCCAGGGACCTCTCTCTAATC	TACAACATGGGCTACAGGCTTG

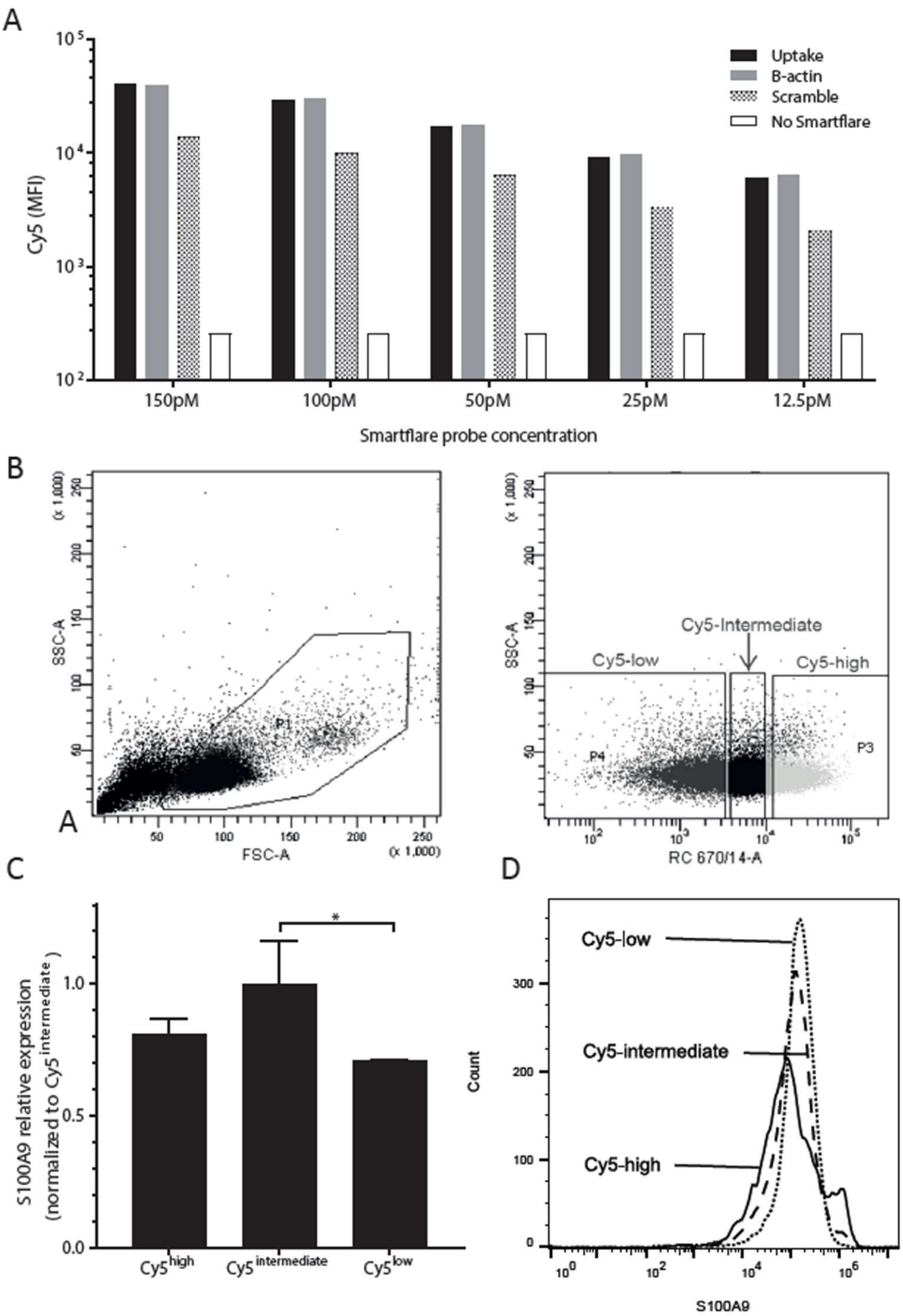

Figure S1. Smart Flare failed to report the mRNA level in live cells. (A) Monocytes were incubated for 16 hours with different amounts of SmartFlare probe. The scrambled control probe produced strong signals, indicating high background levels. (B) Monocytes were sorted into three subsets based on the Cy5 fluorescence intensity: Cy5-high, Cy5-intermediate, and Cy5-low. (C,D) The mRNA (C) and protein (D) level of S100A9 in three FACS-sorted subsets were not consistent with the SmartFlare signals. Flags show means with SD. Differences were tested by one-way ANOVA with Tukey's multiple comparison tests. Data in panel C are shown as means ± SD of three biological replicates. *P<0.05.
